# A deep belief network-based clinical decision system for patients with osteosarcoma

**DOI:** 10.3389/fimmu.2022.1003347

**Published:** 2022-11-18

**Authors:** Wenle Li, Youzheng Dong, Wencai Liu, Zhiri Tang, Chenyu Sun, Scott Lowe, Shuya Chen, Rachel Bentley, Qin Zhou, Chan Xu, Wanying Li, Bing Wang, Haosheng Wang, Shengtao Dong, Zhaohui Hu, Qiang Liu, Xintian Cai, Xiaowei Feng, Wei Zhao, Chengliang Yin

**Affiliations:** ^1^ Department of Orthopaedic Surgery, People’s Hospital of Xinjiang Uygur Autonomous Region, Urumqi, Xinjiang, China; ^2^ Center for Molecular Imaging and Translational Medicine, Xiamen University, Xiamen, China; ^3^ Key Laboratory of Molecular Medicine, The Second Affiliated Hospital of Nanchang University, Nanchang, China; ^4^ Department of Orthopaedic Surgery, the First Affiliated Hospital of Nanchang University, Nanchang, China; ^5^ School of Physics and Technology, Wuhan University, Wuhan, China; ^6^ AMITA Health Saint Joseph Hospital Chicago, Chicago, IL, United States; ^7^ College of Osteopathic Medicine, Kansas City University, Kansas, MO, United States; ^8^ Foundation Program, Newham University Hospital, London, United Kingdom; ^9^ Radiation Oncology, Mayo Clinic, Rochester, MN, United States; ^10^ Clinical Medical Research Center, Xianyang Central Hospital, Xianyang, China; ^11^ Department of Orthopaedics, The Second Hospital of Jilin University, Changchun, China; ^12^ Department of Spine Surgery, Second Affiliated Hospital of Dalian Medical University, Dalian, China; ^13^ Department of Spine Surgery, Liuzhou People’s Hospital, Liuzhou, China; ^14^ Graduate School, Xinjiang Medical University, Urumqi, Xinjiang, China; ^15^ Department of Neuro Rehabilitation, Shaanxi Provincial Rehabilitation Hospital, Xi 'an, China; ^16^ Faculty of Medicine, Macau University of Science and Technology, Macau, Macau SAR, China

**Keywords:** Osteosarcoma, lung metastasis, deep belief networks, prognosis, clinical decision

## Abstract

Osteosarcoma was the most frequent type of malignant primary bone tumor with a poor survival rate mainly occurring in children and adolescents. For precision treatment, an accurate individualized prognosis for Osteosarcoma patients is highly desired. In recent years, many machine learning-based approaches have been used to predict distant metastasis and overall survival based on available individual information. In this study, we compared the performance of the deep belief networks (DBN) algorithm with six other machine learning algorithms, including Random Forest, XGBoost, Decision Tree, Gradient Boosting Machine, Logistic Regression, and Naive Bayes Classifier, to predict lung metastasis for Osteosarcoma patients. Therefore the DBN-based lung metastasis prediction model was integrated as a parameter into the Cox proportional hazards model to predict the overall survival of Osteosarcoma patients. The accuracy, precision, recall, and F1 score of the DBN algorithm were 0.917/0.888, 0.896/0.643, 0.956/0.900, and 0.925/0.750 in the training/validation sets, respectively, which were better than the other six machine-learning algorithms. For the performance of the DBN survival Cox model, the areas under the curve (AUCs) for the 1-, 3- and 5-year survival in the training set were 0.851, 0.806 and 0.793, respectively, indicating good discrimination, and the calibration curves showed good agreement between the prediction and actual observations. The DBN survival Cox model also demonstrated promising performance in the validation set. In addition, a nomogram integrating the DBN output was designed as a tool to aid clinical decision-making.

## Introduction

Osteosarcoma is a malignant bone tumor with a low incidence but a high mortality rate, mainly occurring in children and adolescents (1, 2). Osteosarcoma frequently arises in the extremities, but it is also found in the axial skeleton and can be diagnosed at any age. At present, the combined treatment for Osteosarcoma includes tumor resection, radiotherapy, and chemotherapy. In addition, preoperative neoadjuvant chemotherapy and preoperative radiotherapy are increasingly important (3–5). Despite these advances, the prognosis of patients with Osteosarcoma is still poor due to the aggressive and metastatic behavior of the tumor (6, 7). Previous studies have shown that the major predictors of prognosis include age, gender, tumor dimension, response to chemotherapy, involvement of the proximal extremity or within the axial skeleton, and the presence of metastasis at diagnosis (8, 9). Osteosarcoma can metastasize to distant sites, mainly to the lungs, and occasionally to bone or lymph nodes (10, 11). Metastases can be detected in about 15%-20% of newly diagnosed Osteosarcoma patients, and the 5-year survival rate for these patients with metastases is only 20% (12–14). Therefore, early identification of patients at high risk of metastasis and timely assessment of overall survival is particularly important for reducing mortality and improving patients’ quality of life.

In most situations, researchers conducted survival analyses often used Cox proportional hazards model (15). The model can analyze the influence of multiple factors on survival time simultaneously without estimating the distribution type of survival data, applying survival outcome and survival time as the dependent variable. The model assumes that the risk of a clinical outcome is a linear combination of the patient’s covariates. However, this method may be too crude for many intricate clinical outcomes such as tumor metastasis.

Machine learning (ML) is a significant subfield of Artificial intelligence (AI) to build decision-making models, it concentrates on making predictions by learning from available data. Deep learning (DL) is a sub-field of ML that concentrates on making predictions using multi-layered neural network algorithms. Compared to other ML methods such as logistic regression, the neural network architecture of DL enables the models to scale exponentially with the growing quantity and dimensionality of data (16). The deep belief network (DBN) model is a DL algorithm that stacks simpler models known as restricted Boltzmann machines (RBMs) (17). The unsupervised learning builds a multi-level structure layer-by-layer, automatically extracting more abstract representations from the layers. It makes DBN particularly useful for solving complex computational problems such as large-scale image classification, natural language processing and speech recognition and translation (17).

Recently, numerous ML-based studies have been carried out for cancer prediction, prognosis, or even assessing treatment response (18–20). They predict survival time in years (particularly for the pre-operative prognosis of tumor patients) by using regression or categorizing it into long-term or short-term based on phenotypic features extracted from various types of pre-operative clinical characteristics or image data, i.e., blood tests, computed tomography (CT), magnetic resonance imaging (MRI) data before operation. The ML algorithms used in these studies include Decision Trees (DT), Gradient Boosting Machine (GBM), Random Forest (RF), Naive Bayes Classifier (NBC) and DL. However, few studies have compared the diagnostic performance of various ML algorithms with DL algorithms in assessing lung metastasis in OS patients.

In this study, the performance of DBN and six machine learning algorithms in predicting lung metastasis for Osteosarcoma patients was compared to find the optimal algorithm. The optimal DBN algorithm was subsequently used to construct a pulmonary metastasis predictive model for pulmonary metastasis in Osteosarcoma patients, the predictive model was integrated with other important variables into the Cox model to predict overall survival in Osteosarcoma patients. The study demonstrated that the DBN survival Cox model had good discrimination and calibration, which would provide great help for clinical decision-making.

## Materials and methods

### Applications of deep learning in medical field

DL has developed into an innovative field in the exploration part of ML and data mining (21, 22), which can break through the limitations of human eyes and reveal hidden information (23–25). CNN is a representative deep neural network (DNN) model, which is considered one of the most commonly used DL methods (26), it eliminates the need for tedious steps such as manual feature extraction of medical images and significantly improves the ability to classify images and detect objects in images. CNNs have been gradually used by the medical community to assist in the early diagnosis of clinical diseases and to predict clinical outcomes of disease progression since the introduction of AlexNet in 2012 (27–30). Research shows that deep CNNs can achieve state-of-the-art performance in tumor detection and diagnosis compared to other machine learning methods and human experts (31–33). JY et al. developed and validated a deep learning signal (DLS) from diffusion tensor imaging (DTI) using a deep CNN model, identified key pathways for DLS in a radiogenomics cohort (n=78) from paired DTI and RNA-seq data, and could improve stratification of gliomas by identifying risk groups that affect survival outcomes for dysregulated biological pathways. Gun Woo Lee et al. created a CNN model to diagnose spinal cord cervical spondylosis (CSM) by receiving input from multiple channels of two-dimensional data and performing iterative transformations using convolution and pooling operations to identify features of lateral cervical spine radiographs of patients with or without spinal cord cervical spondylosis (CSM) with high diagnostic accuracy (34). This also provides new ideas and references for the treatment and diagnosis of clinically relevant diseases (35–37).

### Dataset and preprocessing

The training set for this study was derived from the Surveillance, Epidemiology and End Results (SEER) database using the SEER*stat software (version 8.6.3) from 2010 to 2016. Inclusion criteria included: (1) patients with histologically proven osteosarcoma (ICD-0-3 8936/3); (2) the primary tumor had to be localized in the limb bone; and (3) osteosarcoma was the first primary tumor. Patients with missing survival data or data on tumor size, metastasis, stage, or surgical modality were excluded. Collected variables included age, sex, race, grade, primary sites, laterality, bone metastases, T, N stage, lymph node surgery, surgery, radiation, chemotherapy survival status, and survival time. The validation set was collected from the inpatient Electronic Medical Record database at the Second Hospital of Jilin University, the Second Hospital of Dalian Medical University, Xianyang Central Hospital, and Liuzhou People’s Hospital in China. The variables to be collected were the same as before. Time-to-event or censoring was based on the date of diagnosis or the date of the last contact. The Osteosarcoma’s tumor node metastasis (TNM) stage was evaluated based on the 7th edition of the American Joint Committee on Cancer (AJCC) staging manual. “SEER Combined Mets at DX-lung (2010 +)” was used to identify the presence of lung metastasis in a newly diagnosed. Osteosarcoma patient. The Ethics Review Board of the Xianyang Central Hospital approved this study(Ethics Committee number: 20210022).

### Variables screening for overall survival in OS patients

Cox proportional hazards with the least absolute shrinkage and selection operator (LASSO) penalty were employed to identify clinical variables that were linked with the overall survival of Osteosarcoma patients. LASSO penalized estimation methods shrank the estimates of the regression coefficients towards zero relatives to the maximum likelihood estimates. The shrinkage was to prevent overfitting due to either collinearity of the covariates or high dimensionality.

### Deep learning algorithm versus machine learning algorithms

To compare the DBN algorithm with other ML algorithms for pulmonary metastasis in OS patients, several supervised classification methods were evaluated to determine better classification accuracy. The evaluated conventional classifiers include DT, GBM, logistic regression (LR), NBC, RF, and XGBoost (XGB). For parameters identification of six ML algorithms, univariate and multivariate logistics regression analyses were conducted. The preprocessed labelled dataset was used to train and test the model of different classifiers using 10-fold cross-validation as the experimental setting. The 10-fold cross-validation is a method to validate the studied/built model by iterating through the labelled data 10 times with different subsets of training and testing for each iteration.

DBN comprises multilayer random variables and binary la-tent variables and is a probabilistic deep learning algorithm. The model training performed is arranged in two main steps. Step 1: Train each layer of the RBM Network separately in an unsupervised manner and ensure that the maximum feature information is retained when the feature vector is mapped to a different feature space. Step 2: The BP Network is set as the final layer of the DBN, and the output feature vectors of the RBM are used as its input feature vectors to train the entity relationship classifier in a supervised manner.

In this study, the number of attribute features was obtained from the non-negative matrix decomposition as K = 14. After training the DBN, the last layer is the output features. The dimensionality of the feature vector is the number of nodes in the last layer. The number of nodes is determined by parameter sensitivity experiments based on the characteristics of our data. We finally chose 4 as the number of nodes.

In lung metastasis prediction, the attribute vector V of each case sample was used after the dimensionality reduction process as the input of DBN. In this training phase, the input vector V of the visible layer was passed to the hidden layer. Conversely, the input V of the visible layer was randomly selected to reconstruct the original input data. Finally, these new visible neuron activation units forwarded the reconstruction of the hidden layer activation units to obtain h1 and h2. Gibbs sampling was used to repeat the above process during the training. The correlation differences between the hidden layer activation units and the input visible layer were used as the basis for updating the weights W1 and W2.

The above seven algorithms were evaluated by the following indexes: accuracy, precision, recall, and F-measure (F1-Score). In addition, the feature importance of the optimal ML algorithm was calculated and compared with the DBN algorithm.

### Model development and visualization

The previous screening variables selected by the LASSO Cox proportional hazards model were combined with output of the pulmonary metastasis predictive model to construct an integrated Cox model for predicting overall survival in Osteosarcoma patients. A web calculator and a clinical dynamic nomogram for those screening variables were planned to calculate and visualize the relationship between the variables and predicted probabilities of overall survival.

## Model validation

The performance of the DBN survival Cox model was internally validated using the training set and externally authorized using the validation set. Discrimination for the model was evaluated by the area under the curve (AUC) of the receiver operating characteristic (ROC) curve. AUC values can range from 0 to 1, with values of 0.5-0.7 demonstrating poor discrimination, 0.7-0.8 acceptable, and >0.8 excellent discrimination (38). The calibration curve was used to assess the agreement between model-predicted and actual overall survival. The closer the calibration curve is to the 45-degree diagonal, the more perfect the model will be (39).

## Assessment of clinical utility

Decision curve analysis (DCA) was used to explore the net benefit of the prognostic model over the entire range of probability thresholds (40, 41). DCA calculates the clinical net benefit acquired by applying the studied DBN survival Cox model to make clinical decisions. In the plot of DCA, the X-axis indicates thresholds probability for survival, and the Y-axis demonstrates net benefits depending on different thresholds probability. The pink horizontal line parallel to the X-axis represents no clinical action taken by any patient (“treat none”) and their net clinical benefit is 0. The smooth arc line represents that all patients took clinical action (“treat all”), and their net clinical benefit is a backslash with a negative slope. The curve of “treat by DBN survival Cox model” is then compared to “treat all” and to “treat none”.

## Statistical analysis

The baseline categorical variables were represented as their counts and percentage, and continuous variables were expressed as means with standard deviation (SD) and medians [interquartile ranges (IQR)]. The COX proportional hazards with the LASSO penalty were used to identify predictor variables for survival. The lambda (λ) parameters in LASSO regression analysis were chosen for minimized expected model deviance. To compare deep learning and six machine learning algorithms, univariate and multivariate logistic regression analysis were conducted for parameter selection. After a univariate logistics regression analysis of all collected variables, those with a P-value < 0.05 were included in multivariable logistics regression. Variables with P-value < 0.05 in multivariable analysis were used to build the model. Accuracy, precision, recall, and F-measure were used to assess the performance of algorithms. The discrimination and calibration of the model in training and validation sets were evaluated by using the AUC and calibration curve. Clinical applicability was analyzed using DCA. P values < 0.05 indicated statistical significance. The statistical analyses were performed using RStudio software version 1.1.414 (Boston, MA, USA).

## Results

### Data characteristics

**Figure 1:**
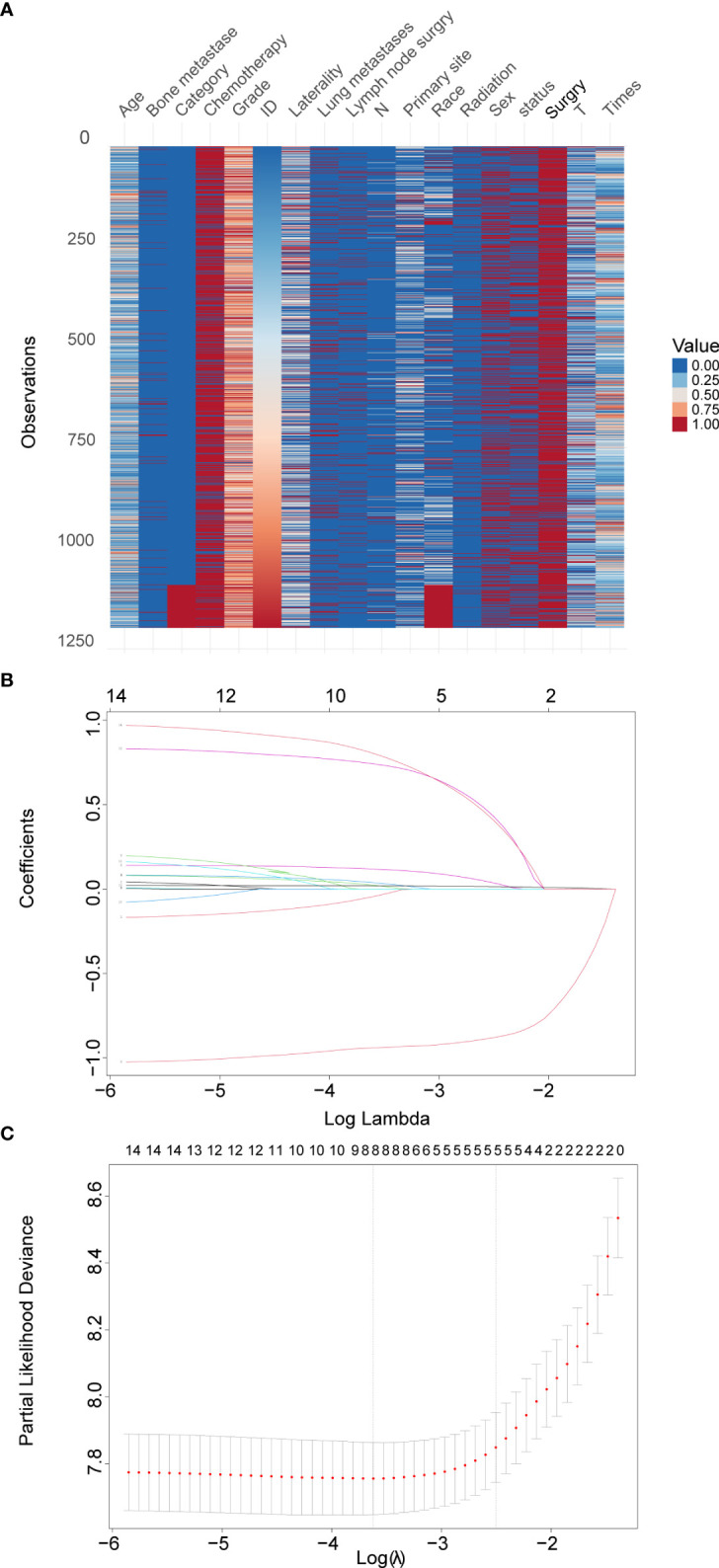
Overview of the total datasets combining the training set and validation set and LASSO model profile plots. **(A)**, Heatmap of each clinical factor in the total datasets. **(B)**, Coefficient profile plots showing how the size of the coefficients of clinical factors shrinks with increasing value of the penalty, with the factors and their regression coefficients selected for the model based on the optimal for the LASSO model. **(C)**, Penalty plot for the LASSO model; color error bars indicate standard error. LASSO, least absolute shrinkage and selection operator.

In total, 1,094 eligible Osteosarcoma patients from the SEER database were identified and designated as the training set. The validation set included 107 patients from the Second Hospital of Jilin University, the Second Hospital of Dalian Medical University, Xianyang Central Hospital and Liuzhou People’s Hospital. An overview of the total datasets combining the training set and validation set is shown in **Figure 1A**.

### Predictor variables of overall survival

Among 14 candidate survival-associated variables, 8 variables with statistically significant hazard ratios were selected based on LASSO Cox regression analysis (**Figures 1B, C**). These were sex, age, primary site, grade, T stage, surgery, bone metastases, and lung metastases.

### Compare with other machine learning classifiers

**Figure 2:**
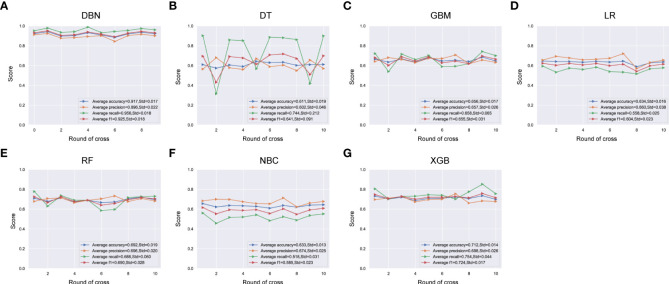
Comparison of DBN **(A)** algorithm and other 6 machine learning algorithms including DT **(B)**, GBM **(C)**, LR **(D)**, RF **(E)**, NBC **(F)** and XGB **(G)** for accuracy, precision, recall, and F-measure (F1-Score) in training set. DBN, Deep Belief Networks; DT, Decision Tree; GBM, Gradient Boosting Machine; LR, Logistic Regression; RF, Random Forest; NBC, Naive Bayes Classifier; XGB, XGBoost.

**Figure 3:**
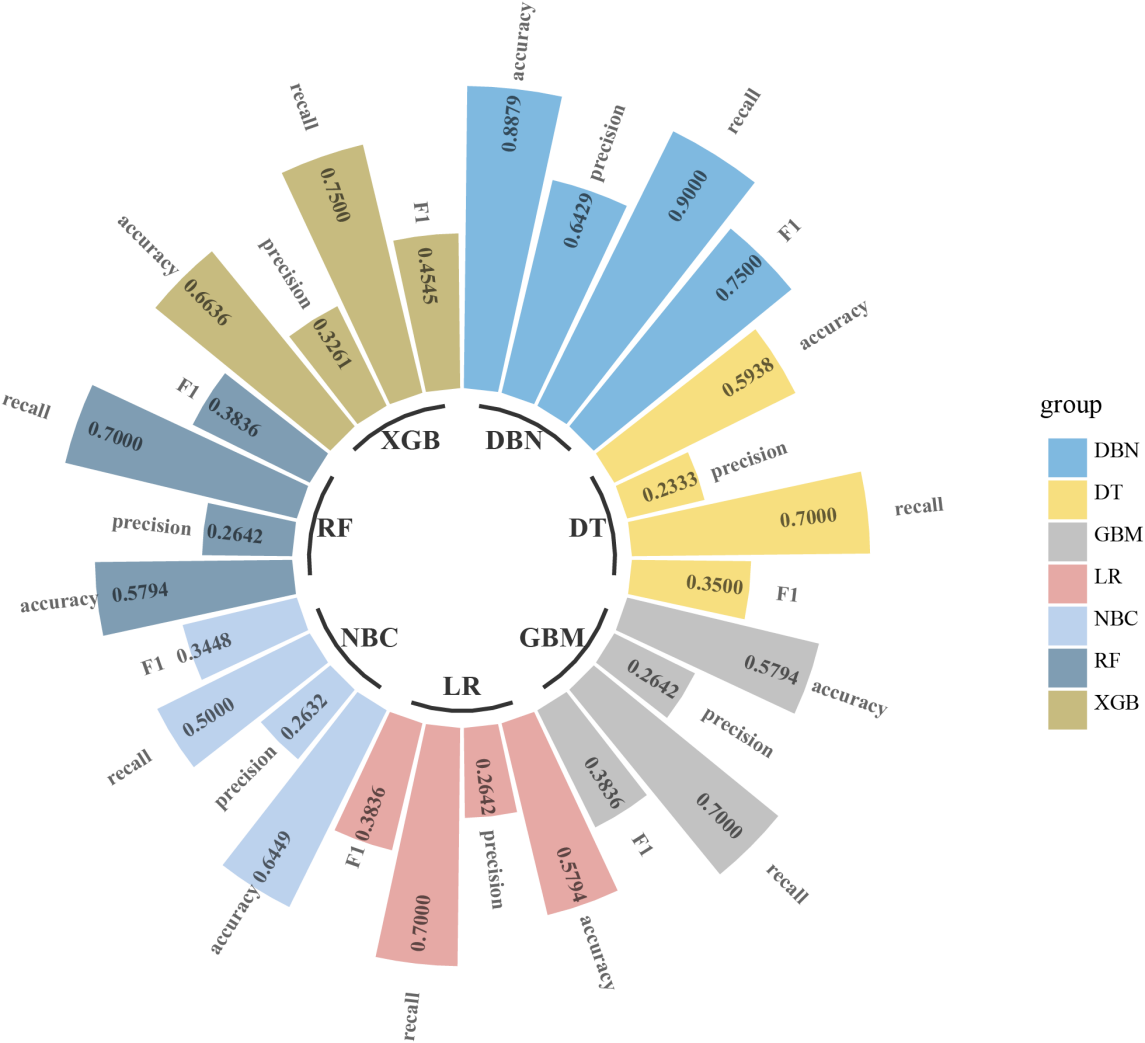
Comparison of DBN algorithm and other 6 machine learning algorithms including DT, GBM, LR, RF, NBC and XGB for accuracy, precision, recall, and F-measure (F1-Score) in validation set. DBN, Deep Belief Networks; DT, Decision Tree; GBM, Gradient Boosting Machine; LR, Logistic Regression; RF, Random Forest; NBC, Naive Bayes Classifier; XGB, XGBoost.

**Figure 4:**
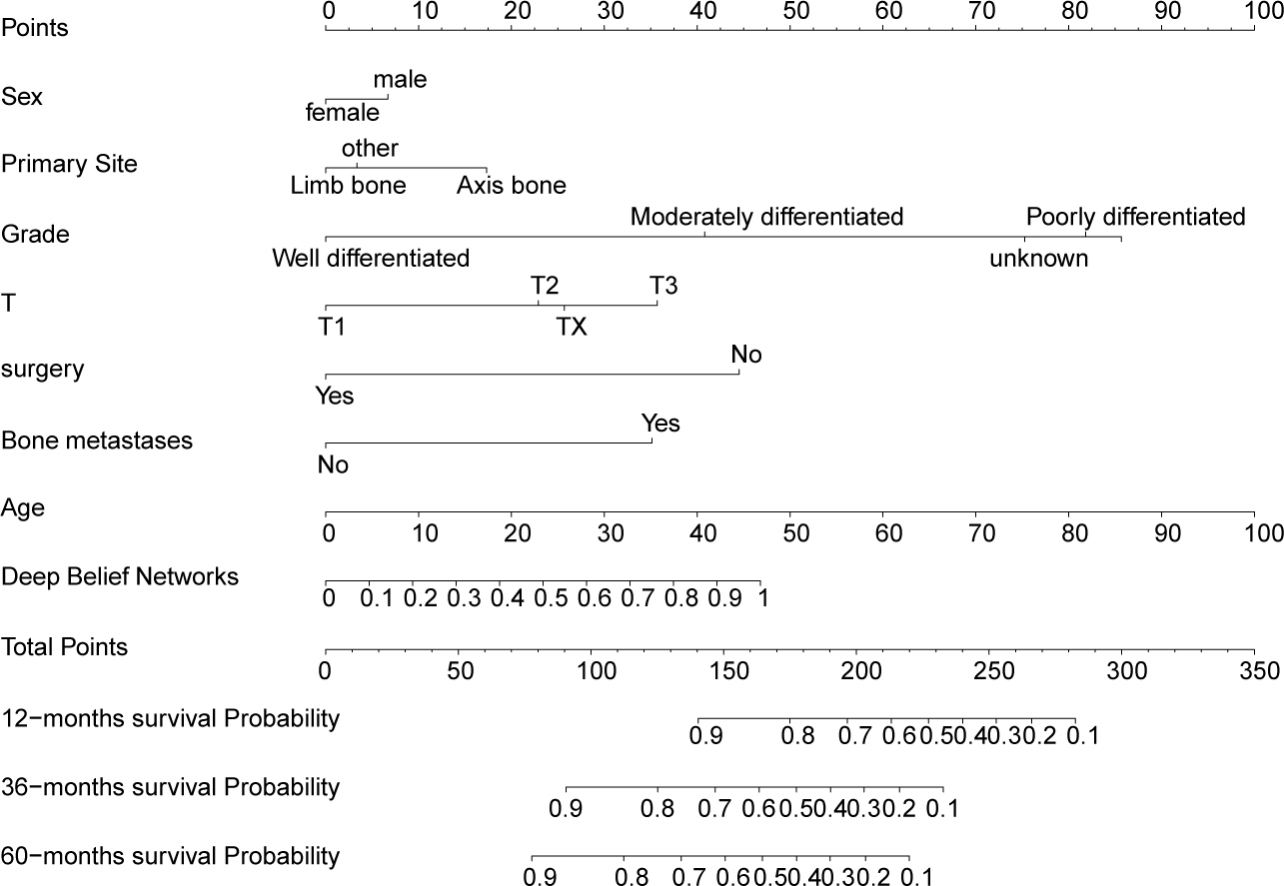
Nomogram for predicting overall survival for 1-, 3-, and 5-years in OS patients. Each variable value for the individuals was determined according to the top Points scale, and then the points for each variable were added. Finally, a personalized survival probability was obtained according to the bottom Total Points scale.

Uni- and multivariate logistic regression analysis identified that sex, bone metastasis, surgery, N and T were significant factors for predicting pulmonary metastasis (**Table 1**). For training set, the results of DBN algorithm and six ML algorithms (DT, GBM, LR, NBC, RF and XGB) are shown in **Figure 2**. The accuracy, precision, recall, and F-Score of DBN were 0.917 ± 0.017, 0.896 ± 0.022, 0.956 ± 0.018 and 0.925 ± 0.018, respectively. Among the six ML algorithms, the accuracy, precision, recall and F- Score of the best algorithm (XGB) were 0.712 ± 0.014, 0.689 ± 0.026, 0.754 ± 0.044 and 0.724 ± 0.017, respectively. For the validation set, the comparison result is shown in **Figure 3**. The accuracy, precision, recall, and F-Score of DBN and XGB were 0.888/0.665, 0.642/0.326, 0.900/0.750 and 0.750/0.455, respectively. **Figure 4** shows the ranking of the top 12 feature importance according to the DBN and the XGB algorithm. As shown, ‘N stage’ and ‘T stage’ was the most two important predictors of lung metastasis in the DBN algorithm. ‘Surgery’ was the most important predictor, and ‘T stage’ was the second most important predictor of lung metastasis in the XGB algorithm.

**Table 1:** Univariate and multivariate Logistics regression for pulmonary metastasis of osteosarcoma.

Characteristics	Univariate logistics regression			Multivariable logistics regression		
	OR	CI	P	OR	CI	P
Age	1	1-1.01	0.637	NA	NA	NA
Bone metastases						
No	Ref	Ref	Ref	Ref	Ref	Ref
Yes	8.58	4.92-14.96	<0.001	5.29	2.88-9.73	<0.001
Chemotherapy						
No	Ref	Ref	Ref	Ref	Ref	Ref
Yes	1.48	0.99-2.22	0.058	NA	NA	NA
Grade						
Well differentiated	Ref	Ref	Ref	Ref	Ref	Ref
Moderately differentiated	3.61	0.4-32.75	0.254	NA	NA	NA
Poorly differentiated	6.39	0.85-48.03	0.072	NA	NA	NA
Undifferentiated; anaplastic	5.95	0.8-44.32	0.082	NA	NA	NA
unknown	5.11	0.68-38.58	0.113	NA	NA	NA
Laterality						
Left	Ref	Ref	Ref	Ref	Ref	Ref
Right	1.13	0.83-1.56	0.431	NA	NA	NA
Other	0.84	0.51-1.37	0.475	NA	NA	NA
Lymph node Sur						
No	Ref	Ref	Ref	Ref	Ref	Ref
Yes	0.56	0.33-0.95	0.031	0.83	0.47-1.45	0.512
N						
N0	Ref	Ref	Ref	Ref	Ref	Ref
N1	2.61	1.28-5.31	0.008	1.54	0.67-3.57	0.31
NX	2.82	1.72-4.62	<0.001	2.06	1.17-3.62	0.012
Primary Site						
	Ref	Ref	Ref	Ref	Ref	Ref
Primary Site1	0.82	0.58-1.17	0.271	NA	NA	NA
Primary Site2	0.81	0.46-1.43	0.471	NA	NA	NA
Race						
White	Ref	Ref	Ref	Ref	Ref	Ref
Black	1.12	0.72-1.72	0.62	NA	NA	NA
Other	1.08	0.73-1.59	0.7	NA	NA	NA
Radiation						
No	Ref	Ref	Ref	Ref	Ref	Ref
Yes	1.74	1.16-2.6	0.007	1.38	0.87-2.19	0.174
Sex						
Male	Ref	Ref	Ref	Ref	Ref	Ref
Female	0.63	0.46-0.85	0.003	0.57	0.41-0.79	0.001
Surgery						
No	Ref	Ref	Ref	Ref	Ref	Ref
Yes	0.27	0.2-0.38	<0.001	0.39	0.27-0.58	<0.001
T						
T1	Ref	Ref	Ref	Ref	Ref	Ref
T2	2.56	1.73-3.78	<0.001	2.46	1.63-3.71	<0.001
T3	6.03	2.93-12.41	<0.001	4.29	1.94-9.52	<0.001
TX	3.58	2.23-5.73	<0.001	2.23	1.31-3.77	0.003

OR, odds ratio; CI, confidence interval; Ref, reference; NA, not available.

### Model development

**Figure 5:**
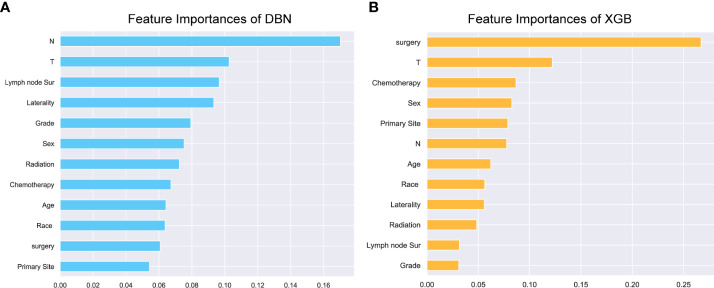
Feature importance. The top 12 feature importance results for the DBN **(A)** model and XGB **(B)** model trained on training set. DBN, Deep Belief Networks; XGB, XGBoost.

The variables of sex, age, primary site, grade, T, surgery, and bone metastases were combined with the output of DBN-based lung metastasis predictive model to construct an integrated DBN survival Cox model for predicting overall survival in OS patients. To make the DBN survival Cox model more intuitional, a nomogram (**Figure 5**) was then constructed. An online web calculator embedding a dynamic nomogram with our DBN survival Cox model was also developed, which is available at https://drwenleli0910.shinyapps.io/ODSapp/. After filling in the online form as required in the webpage, the webpage will automatically generate a personalized nomogram, together with the probability of survival at 1-, 3-, and 5-years.

### Performance of the prediction model

**Figure 6:**
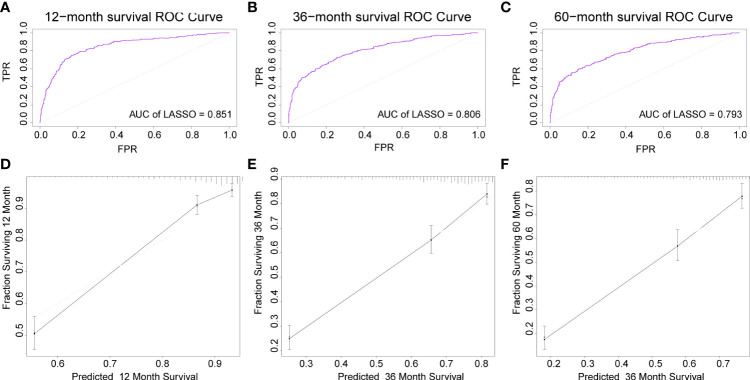
The AUC and calibration curves of DL survival Cox model at 1-, 3-, 5-years in the training set. **(A–C)**, the AUC of the model at 1-, 3-, 5-years. **(D–F)**, the calibration curves of model at 1-, 3-, 5-years. AUC, area under the curve; DL, deep learning.

**Figure 7:**
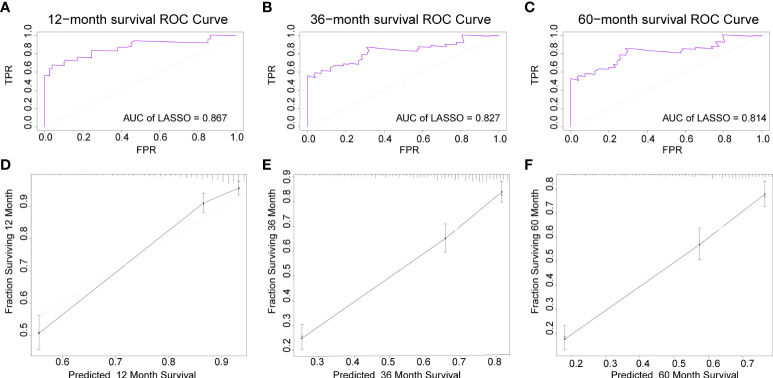
The AUC and calibration curves of DL survival Cox model at 1-, 3-, 5-years in the validation set. **(A–C)**, the AUC of model at 1-, 3-, 5-years. **(D–F)**, the calibration curves of model at 1-, 3-, 5-years. AUC, area under the curve; DL, deep learning.

**Figure 8:**
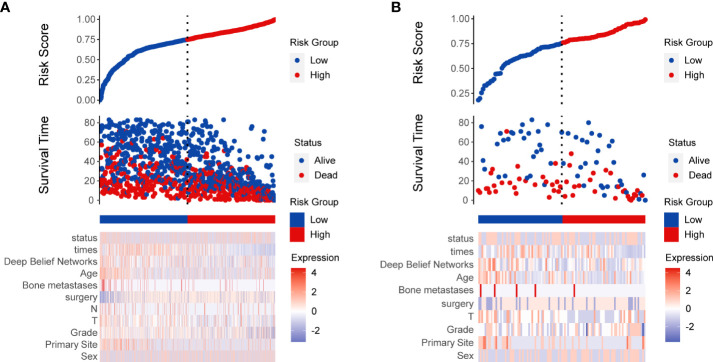
Risk score analysis of deep learning survival Cox model in training **(A)** and validation **(B)** set.

The AUC of the DBN survival Cox model at 1-, 3-, 5-years in the training set were 0.851, 0.806, and 0.793, respectively (**Figures 6A–C**). Calibration curves showed that the predicted and actual survival rates matched very well at 3 and 5 years, and were acceptable at 1-year (**Figures 6D–F**). In the validation set, the AUC of DBN survival Cox model at 1-, 3-, 5-years were 0.876, 0.827, and 0.814, respectively (**Figures 7A–C**). The predicted and actual survival rates matched very well at 1-year, and were accepted at 3- and 5-years (**Figures 7D–F**). The risk curve, survival status and time, and expression of features in the DBN survival Cox model were also represented according to the high- and low-risk groups in the training and validation set (**Figure 8**).

### Assessment of clinical utility

**Figure 9:**
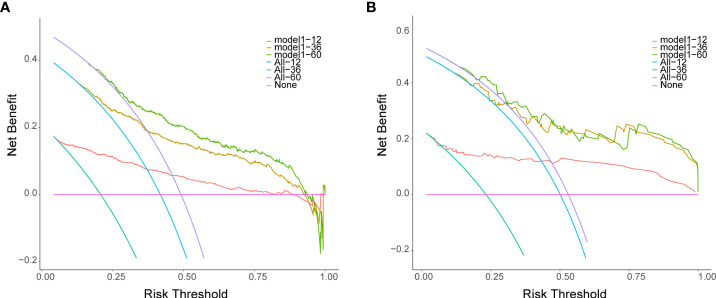
Decision curves for the DL survival Cox model for 1-, 3-, 5-years in the training set **(A)** and validation set **(B)**. The X-axis indicates thresholds probability for lung metastasis risk, and the Y-axis indicates net benefits depending on different thresholds probability. The pink horizontal line parallel to the X-axis represents no clinical action taken by any patient (“treat none”) and their net clinical benefit is 0. The smooth arc line represents that all patients took clinical action (“treat all”), and their net clinical benefit is a backslash with a negative slope. The curve of “treat by DL survival Cox model” is then compared to “treat all” and to “treat none”.

The decision curves for 1-, 3- and 5-years in the training set and validation set (**Figures 9A, B**) demonstrated relatively good performance for the DBN survival Cox model in terms of clinical application. As shown, when the threshold probability in the clinical decision was more than 0.063, 0.151, and 0.193 for 1 year, 3 years, and 5 years, respectively, the DBN survival Cox model provided more of a net benefit than “ treat all “ or “ treat none”.

## Discussion

Although the effectiveness of the localized Osteosarcoma treatment has gradually improved, the 5-year overall survival of Osteosarcoma patients with lung metastasis is less than 30%, suggesting that these patients still fare poorly (12–14). Several studies have explored potential risk factors for tumor metastasis to facilitate early management (8, 9). However, these articles mainly concentrated on the impact of a single factor on metastasis, a study on the combined impact of multiple factors on metastasis is still lacking.

Cox proportional hazards model is the traditional statistical approach for survival analysis and risk prediction. The data in this model are required to meet prior assumptions, including the proportional hazard assumptions and linear relationships between continuous covariates and the log hazard function.

However, in the complex biomedical field, there are many nonlinear relationships (42, 43). In this context, a single Cox model with proportional hazards is likely to produce less accurate estimations of survival outcomes. Therefore, new solutions containing these potentially nonlinear variables are highly needed to accurately predict the survival of individuals,

DBN, a relatively new computational algorithm that has become a popular research topic, has been rapidly developed and widely used in medical research. It is a DL-based generative model, when a network contains a large number of deep layers, which addresses the problem of vanishing gradients that suffer from traditional gradient-based learning algorithms. In cancer research, the advantages of DBN model for survival analysis are as follows: First, this model shows a modified fit for features with a nonlinear relationship, which applies to the nonlinear associations that are abundant in real-world practice. Second, as a DL model, the DBN automatically learns complex mapping by transforming the features through the multi-layer structure. At present, most ML algorithms have applied shallow-structured architectures.

These models are specifically effective in solving well–constrained problems. However, several studies have confirmed reasons for using deep structures (44–46). Deep models may be more robust in the wide variety of functions that can be parameterized by composing non-linear transformations (47). They also allow more efficient representation of highly varying functions than shallow architectures. In addition, a prominent problem with traditional algorithms is the requirement for a certain level of domain expertise to design a feature extractor that converts raw data into a suitable feature vector (16). DBN allows a system to be fed with raw data and to automatically discover the required representations. This study shows that the DBN-based model can achieve better prediction performance than other machine learning algorithms.

Our predictive models currently use seven clinical variables, including common demographic and clinical characteristics and histopathological outcomes. The SEER database was used with relatively complete data during the model development, while some data were missing in the external validation set. Our model has tolerance for missing data, and we still achieved high performance on the external validation set even missing 30% data of the patients. In addition, a dynamic nomogram can provide dynamic assessments as missing data are supplemented during subsequent treatment.

There were aols some limitations despite the promising results. First, the training set was extracted from the SEER database with a relatively small sample size, although an independent validation group from various hospitals was used. Thus, a large sample of multicenter data was required to fully evaluate the generalization ability of the DBN survival COX model. Second, the SEER database only provided limited clinical variables, and many variables closely associated with tumor metastasis and survival, such as tumor markers and gene expression, were not available. Future studies could incorporate these potentially essential factors and construct a more comprehensive predictive model.

## Conclusion

In our study, a DBN survival Cox model was established to predict the overall survival in Osteosarcoma patients. Compared to the other six ML algorithms, this DNB algorithm demonstrated better performance. Both internal validation and external validation revealed good generalizability. In addition, an individualized risk estimate of survival can be calculated through the nomogram and online web calculators developed by this study. This allows the model to be applied to clinical practice and helps with clinical decision-making.

## Data availability statement

The original contributions presented in the study are included in the article/supplementary material. Further inquiries can be directed to the corresponding authors.

## Author contributions

CLY, WZ and XWF completed the entire research design. WLL, YZD and WCL participated in the research and analyzed data. WLL and YZD drafted manuscripts. CYS and SL provided expert consultation and advice. SYC, RB, QZ, CX, WYL and BW participated in its design and coordination, HSW, STD and ZHH helped polish the language. All authors reviewed the final version of the manuscript.

## Conflict of interest

The authors declare that the research was conducted in the absence of any commercial or financial relationships that could be construed as a potential conflict of interest.

## Publisher’s note

All claims expressed in this article are solely those of the authors and do not necessarily represent those of their affiliated organizations, or those of the publisher, the editors and the reviewers. Any product that may be evaluated in this article, or claim that may be made by its manufacturer, is not guaranteed or endorsed by the publisher.
